# Endurance, resistance and resilience in the South African health care system: case studies to demonstrate mechanisms of coping within a constrained system

**DOI:** 10.1186/s12913-015-1112-9

**Published:** 2015-09-29

**Authors:** John Eyles, Bronwyn Harris, Jana Fried, Veloshnee Govender, Pascalia Munyewende

**Affiliations:** Centre for Health Policy, School of Public Health, Faculty of Health Sciences, University of the Witwatersrand, 27 St Andrews Road, Parktown, 2193 Johannesburg, South Africa; Centre for Agroecology, Water and Resilience, Coventry University, Priory Street, CV1 5FB Coventry, UK; Health Economics Unit, University of Cape Town, School of Public Health and Family Medicine, Observatory 7925, Cape Town, South Africa

**Keywords:** South Africa, Endurance, Resilience, Resistance, Coping mechanisms, Adaptive behaviour

## Abstract

**Background:**

South Africa is at present undertaking a series of reforms to transform public health services to make them more effective and responsive to patient and provider needs. A key focus of these reforms is primary care and its overburdened, somewhat dysfunctional and hierarchical nature. This comparative case study examines how patients and providers respond in this system and cope with its systemic demands through mechanisms of endurance, resistance and resilience, using coping and agency literatures as the theoretical lenses.

**Methods:**

As part of a larger research project carried out between 2009 and 2010, this study conducted semi-structured interviews and observations at health facilities in three South African provinces. This study explored patient experiences of access to health care, in particular, ways of coping and how health care providers cope with the health care system’s realities. From this interpretive base, four cases (two patients, two providers) were selected as they best informed on endurance, resistance and resilience. Some commentary from other respondents is added to underline the more ubiquitous nature of these coping mechanisms.

**Results:**

The cases of four individuals highlight the complexity of different forms of endurance and passivity, emotion- and problem-based coping with health care interactions in an overburdened, under-resourced and, in some instances, poorly managed system. Patients’ narratives show the micro-practices they use to cope with their treatment, by not recognizing victimhood and sometimes practising unhealthy behaviours. Providers indicate how they cope in their work situations by using peer support and becoming knowledgeable in providing good service.

**Conclusions:**

Resistance and resilience narratives show the adaptive power of individuals in dealing with difficult illness, circumstances or treatment settings. They permit individuals to do more than endure (itself a coping mechanism) their circumstances, though resistance and resilience may be limited. These are individual responses to systemic forces. To transform health care, mutually supportive interactions are required among and between both patients and providers but their nature, as micro-practices, may show a way forward for system change.

## Background

The South African constitution binds the state to work towards the progressive realisation of the right to health. Yet twenty years after democracy, the country is still grappling with massive health inequities. Changes have occurred over the last years as described in the Lancet 2009 series on health in South Africa, which highlighted the challenges within the health system and the country’s quadruple disease burden of HIV, tuberculosis (TB), violence and injury, and non-communicable disease. As Mayosi et al. [1] report in 2012, there have been improvements in leadership, greater linking of tackling HIV and tuberculosis (TB), and recognition of the need for better integration of all health care services to positively influence the social determinants of health. But challenges remain with a growing non-communicable disease burden, unmet Millennium Development Goals (MDGs), difficulties in coordinating and strengthening the health system and a still weak information and surveillance capacity (see [2–5]). The South African Human Rights Commission’s inquiry into the accessibility of health care services [6] noted that quality and availability of care, identifying poor staff attitudes, and inadequate staff levels remain significant challenges at all levels of the health system. The government recognizes the importance of these systemic changes and the need to enhance competencies in the health care as well as other sectors, much of this being enshrined in the national development plan [7]. Thus while the broad parameters of service delivery challenges are known, little is currently known on the coping mechanisms patients and providers use to make do in these circumstances. Knowing these mechanisms is vital as new policies are introduced at the national, provincial and district levels

The purpose of this paper is to explore how individuals (i.e. patients and healthcare providers) manage a challenging and challenged system to obtain service (i.e. patients) and provide it (i.e. healthcare providers) in a meaningful way. Through the use of case studies, we provide a commentary on how providers and patients as individuals cope with these systemic and entrenched challenges. An emergent question is how does their agency become utilized? In tackling agency, we contribute to an existing body of South African literature. Mangcu [8], for example, argues that South Africa has strong large-scale institutions, such as political parties and national government agencies but weak small-scale ones, especially at the level of local governance, within municipalities and wards. Alexander suggests, therefore, that coping must often be at the individual level rather than communal one. Communal institutions and practices do exist but most are linked to ineffective municipal service delivery (housing, power, water) rather than health care (see [9]). Where they exist, as in the form of community health workers or home-based carers, there are often localised schemes that, despite national initiatives, have not yet become widespread, or are in the process of being rolled-out. Additionally, many reforms to transform primary health care (PHC) with an emphasis on more effective human resources are in their early stages, as locally piloted district, ward- or school-based interventions [10, 11]. Yet as Ataguba and Alaba [12] comment, South Africa’s neo-liberal, individualized ways of operating have reinforced health inequities and poor access to services. Furthermore, coping involves interpersonal relationships and the bases of these – in work, between different classes of people – are rooted in historical context. Asymmetries of power derive from apartheid and neo-liberal agendas. Work places are often hierarchical and tense and work itself has itself become precarious (see [13]). For women, e.g. mostly nurses and pharmacy assistants– this is worsened by gender hierarchies in their personal and professional spaces.

There has been much work on how individuals (patients and providers) and communities cope with or navigate stressful, adversarial situations [14, 15]. There is some work on health care providers, mainly around coping with job or work stress. Lee [16] showed how PHC nurses used active – being organized, prioritize tasks, help others – and passive – tolerant, relaxed, communicative – strategies (see [17, 18]). Frontline providers often act in these ways regardless of formal policy or managerial expectations.

Most studies of patient coping behaviour have been concerned with how they cope with a disease or illness. In South African terms, that is often with much family and community support [19, 20]. Few studies have been undertaken with respect to how patients cope with the health care system itself except for them to gain more knowledge and become more communicative with providers. Here, the idea of the ‘sick role’, especially in such paternalistic systems as South Africa’s, remains useful as it suggests a passive and compliant individual. Yet the sick role is seen as “increasingly contingent, constituted by diagnostic procedures and agreed-upon conventions negotiated and renegotiated outside the patient’s body” [21]. So coping is about negotiation, and “only a person who is kept totally confined and controlled does not participate in the dialectic of control” ([22], p39). Coping for the patient contains within itself the power to resist. In this light, coping is more than dealing with stress (see [23]): it can also celebrate resistance and the power of agency. This, too, can apply to providers if they can respond with confidence and re-affirmation of their roles and identities. Coping is then more than acquiescence or making do, it can be positive and assertive, characteristics which this paper is keen to expose.

As negotiation, coping requires agency. Agency may be seen as ‘life conduct’ (Lebensfuhrung) in which individuals respond to and cope with demands and opportunities in an active way [24]. But context matters and as Jungar and Oinas [25] comment, by becoming agents, individuals do not necessarily cease being passive victims. Instead, discourses of agency and victimhood both work towards the production and insertion of autonomous individuals (as agents and victims) within neo-liberal hierarchies of power. This notion that individualised agency and victimhood represent opposite sides of the same neo-liberal coin [25] makes responses to being sick, including whether and how care is sought and delivered, quite complex. Victimhood may remain if everyday life is reproduced. But it can also be transformed if individuals use their networks, resources and personal attributes to resist and become resilient [26]. What capabilities do people have to cope and adapt [27]? In other words, how do individuals act to meet their goals, given contextual and structural forces [28]? Adaptation often takes the form of passivity, especially in the South African public health system known for its often hierarchical and inefficient operations. But for some, potential exemplars for change, coping can manifest as resistance (challenges to the ways in which the system operates and conspires against the agency of patients and providers [29]) and/or resilience (the ability to respond positively to adversity and crises [30]). Both depend on the availability of resources or assets such as human capital (ability to work, health and knowledge), social capital (networks, groups and trust), natural capital (land, water and wildlife), physical capital (transport, shelter and energy) and financial capital (savings, credits). Obrist et al. [31] identify these with respect to mitigation and environmental change but the social, economic, symbolic and cultural (often religious) asset bases shape the possibility and form of resilience. Resistance can take the form of micro-practices which ensure survival or continued assistance and may help individuals endure a claustrophobic system or sets of relationships (see [32]). If society or the health care system acts regressively against those with little power, resilience must be treated with some circumspection as the practices of resilience may have adverse consequences elsewhere. But resilience holds within itself a desire to overcome challenges to the way of life [33] or treatment regime/practice, to go beyond endurance. Thus once agency and coping as negotiation are recognized, individuals are not victims but the recognition of victimhood, and its vulnerabilities, points, in our view, to the centrality of endurance in the living of lives affected by straitened times and with chronic diseases. Endurance – continuing with life and activities – occurs because of the containing system in which people live and from which there is little chance of escape. It has been identified in conflict settings with the disadvantaged being more than victims because of their hope for the future [32, 33]. Such endurance means dealing with stigma, frail bodies, unsympathetic carers, little food and relatively high transport costs to facilities. Such endurance means overcoming being classed as a victim and dealing with vulnerabilities, not only hoping for a better future but also being resilient and resistant in the present [30, 33]. We therefore assert that resistance, resilience and endurance are key mechanisms of coping, involving that based on emotional responses and problem-solving activities (expressions of agency), as well as passivity. These distinctive aspects of coping (and the assets that shape it) are revealed and theorized in these case studies.

Endurance, resilience and resistance may be seen as adaptations to an often faceless expert system and as part of the everyday practices of patients and providers. For this purpose, four individual cases have been selected to illustrate these narratives In this way, their stories can be told more fully than if the entire qualitative sample was used, although that and observations in health care facilities form part of the data context of the analysis. Thus, ways in which patients and providers cope with a resource-poor system, committed to equity but unable to deliver as quickly as needed, are identified. Through these actions, individuals cope through the use of agency.

## Methods

### Setting and data

This research is part of a five-year research study on equity in access to health care (the REACH project) that included both quantitative and qualitative data collection strategies in several provinces of South Africa. This paper draws on the qualitative interview materials. In-depth interviews were conducted with 45 patients ( maternal care services – 16 patients; TB and ART - 29 patients accessing tuberculosis (TB) treatment and antiretroviral (ARV) therapy) and 67 providers from twelve health facilities in three health sub-districts in South Africa – two urban (Cities of Cape Town (Western Cape) and Johannesburg (Gauteng) and one rural (Bushbuckridge, Mpumalanga). In addition, we also conducted observations of the day-to-day practices in the twelve facilities. These facilities were selected from a larger number of health care institutions with the aim to include different facility sizes, management styles as well as geographical and community characteristics. We considered access to three tracer treatments - TB, Antiretroviral Therapy (ART) and maternal deliveries - because they deal with complex, conditions that require ongoing or sustained engagement with the health system. Patients and providers were selected from these facilities based on their availability in clinics and hospitals. Researchers set up meetings in advance with maternal delivery, ART and TB providers and requested a chance to talk with patients attending these services. The intention was to interview patients twice but that proved quite difficult as cell phone numbers change and people are very busy. Patient interviews took the form of their telling about their lives and the role of illness or pregnancy in those lives. The interviews were conducted by trained fieldworkers (mostly women, with some previous knowledge of research interviews and all black) in the language of preference of the respondent and lasted from 30–90 min. Provider interviews were carried out in English and charted the life and career histories of informants and lasted again from 30 to 90 min. For this paper, we select case accounts or specific narratives from four individuals, an approach supported by the qualitative research design literature [34].

The data were collected between June 2009 and July 2010. Ethical clearance was granted by the Universities of Cape Town (Health Sciences Faculty Research Ethics Committee: 460/2008) and Witwatersrand (Human Research Ethics Committee (Medical): R14/49/2008); and the Western Cape Health District Health Services and Programme (19/18/RP11/2008), Gauteng Directorate of Policy, Planning and Research (06/06/2008), and Mpumalanga Health Research and Ethics Committee (MP/09/08). Study permission was also received from the Cities of Johannesburg (23/07/2008) and Cape Town (13/12/2008) and verbal approval was granted by district managers in each study site, as well as heads of the health facilities involved. Informed, written consent was obtained from all individuals interviewed. All interviews were anonymized and stored on a secure server available only to the research team. Pseudonyms were assigned to people and facilities to protect confidentiality. But for this commentary, we emphasize a case approach, using four respondents for analysis, supported by some commentary from others in the discussion section.

To select the cases, the intersection of biography, history and society is important (see [35]). Case stories are selected in which “people strive to configure space and time, deploy cohesive devices, and reveal identity of actors and relatedness of actions across scenes. They create themes, plots, and drama. In so doing, narrators make sense of themselves, social situations, and history” ([36], p iii). Patient case stories must reveal “deeply historicized and social view[s] of health and illness” ([37], p322). We assert the same applies to providers’ stories. Their chosen cases also help to emphasize identities. Individuals thus position themselves in the context of the organizations, cultures and relationships in which they find themselves. Stories are of course performances (see [38]) but the interpretation here is primarily cultural (see [39]) and aims to recognize that illness and its treatment mesh with lives and life’s ambitions [40]. The four cases do reveal meanings yet as Mishler [41] notes we continually re- story our pasts, shifting the relative significance of different events for whom we have become, discovering connections we had previously been unaware of, repositioning ourselves and others in our networks of relationships [42]. By concentrating on specific cases, a feel for sense-making emerges whereby individuals engage in retrospective and prospective thinking in order to construct an interpretation of reality located in context [43]. These senses and meanings emerge in conversations but the ways we interpret must be theoretically grounded (see [44]), permitting simultaneously a deep understanding of the stories within a mindfulness of other work and vitally of the care system in which they are located. We therefore theorize ‘through’ these cases while empirically grounding the meanings and contexts of which they speak.

In analysing these and all interviews, at least two and up to five researchers read and identified key themes and reached agreement. Endurance, resistance and resilience emerged as important themes when reading, coding, and re-reading the breadth of our interviews and observations. This paper uses specific narratives as cases of dealing with health care interactions in a resource-limited setting to reveal how patients and providers cope and navigate through and in the system. This selection of specific cases and narratives has been undertaken for several decades in the social sciences. Morse and Field [43] put forward the idea that the selection of cases is in part based on how individual storytellers connect interpretation (resistance-resilience-endurance) in their texts. As Crowe et al. [44] point out, the bringing together of narrative and case study/selection is a powerful tool as similar questions are asked of all respondents but some present a unique perspective on the interpretation at hand within the given social context [45]. Thus our four cases, supported by some other voices, were purposefully selected on this basis so as to explore an event or phenomenon in depth and in its natural context [46].

The four cases provide narratives revealing real-life situations and their multiple wealth of details, support the argument that coping through endurance, resistance and resilience cannot be meaningfully understood as simply rule-governed acts (see [47]). The individual is specified as the unit of analysis, specification being necessary to understand how the case relates to context and a broader body of knowledge [48] with fewer cases giving the opportunity for depth of observation (see [49]). Hence, while we encountered many instances of endurance, resistance and resilience among patients and providers, the narratives of four individuals were chosen as providing particularly rich information on forms of passivity, and emotion- and problem-based coping with health care interactions in difficult settings. These individuals were selected and screened so as to offer insights into these research themes (see [50]). But as Geertz [51] said that “The Field” itself is a “powerful force: assertive, demanding, even coercive” so that the political and research contexts are vital for interpretation. Thus stories can be dense yet meaningful providing windows on a complex world [35].

## Results

### The cases

In this section, we present four individual cases to demonstrate endurance, resistance and resilience as coping mechanisms, or responses to the challenges faced by receivers and providers of care in South Africa’s public health care system. We introduce patients Sthembiso Nene (young mother) and Thulani Zondo (male TB patient) as well as providers Nomathemba Mxeke (female social worker) and Grace Themba (female pharmacy assistant). These detailed cases allow us to present more than just individual quotes often cited in isolation from the context of individuals’ lives - and give us the opportunity to emphasise the complexity of lives in which endurance, resilience and resistance emerge as important coping mechanisms. Hence, we do present some detail of interviewees’ lives but limit this nonetheless to maintain anonymity of individuals.

### Sthembiso’s story: resilience with few resources

Sthembiso Nene (all names are pseudonyms) is a 20-year old grade-12 pupil who, most of the time, is the sole person responsible for her two younger sisters. Both her parents are alive but mainly absent from the home. Relationships with her parents are strained. The three girls do not have to pay rent but share a single room without electricity or running water. The interview with Sthembiso was conducted a few days after she had given birth.

Given the limited involvement of Sthembiso’s parents, the siblings, who are all still going to school, face very challenging economic conditions that were for example expressed in difficulties in funding Sthembiso’s fare for a hospital visit. For the three sisters, emotional and economic support from outside their family has heightened their resilience:

“We were given food by the neighbours. There is this other woman – she is not here at the moment – she stays in those houses at the back. She is the one who always helped us. The other one stays at that big house. Those are the two people who always helped us. Even the others were helping us, but these two were always there.”

Sthembiso went for an early pregnancy test. Given her fear of bringing a child into the world only to suffer, she contemplated having an abortion, considered controlling her body as a form of resistance. Her boyfriend appeared somewhat supportive in the beginning, but later in her pregnancy seemed to completely ‘disengage’ from their relationship. Given her uncertainty about what to do, Sthembiso – over a long period of her pregnancy – was in denial about its reality, both to herself and others including her sisters. In some instances, she appears extremely vulnerable, a victim of her challenging circumstances. Asked, why she did not go to the clinic earlier on in her pregnancy, she explained:

“You know, the reason why I didn’t go is that I was scared that when I go there they will judge me and shout at me on top of that. The other thing was that I thought they would tell me that I’m still young and I’m having sex already. You know, people have a tendency to judge without understanding what led you to doing what you did. (…) It was from the stories I heard from people, somebody will tell you about their experience at the clinic when they were told that they are young but sleeping with men. So I thought that maybe with me it was going to be worse.”

Sthembiso tried to limit her exposure to possible adverse community reactions. So to avoid public sanction, she endured her condition with little help, displaying a kind of lethargy and passivity to her condition, community and the health care system. In the seventh month she took more serious steps towards getting an abortion, including visiting a clinic (too late) and getting money from her closest friend for a back-street abortion. But she changed her mind since “it is a huge sin to kill an innocent child, (and being also worried that) this is the only child that God is giving (her)”. She thus resisted this way forward because of her religious beliefs, which strengthen her ability to cope [52], and went instead, to a nearby clinic for antenatal care and to book her delivery. Sthembiso did not want to appear ignorant about the need for timely antenatal care, so she lied to clinic staff about the timing of her pregnancy, leading nurses to suspect a twin pregnancy and hence referring her to the hospital for clarification. Due to difficulties in arranging travel fare money in time, she went one day after she was supposed to visit the hospital. Hospital staff then refused to attend to her, insisting that patients referred from her clinic were only attended to on one specific day of the week. After this experience, Sthembiso gave up on trying to book a delivery bed and ended up delivering at home. She was eventually helped by her sister and neighbours, after enduring most of her labour alone. Thus, the resistance of hospital staff to adapt their practices to patients’ needs, financial challenges and Sthembiso’s own anxieties - including an expectation (based largely on hearsay) of bad treatment by providers for returning to the clinic without regularly attending antenatal care nor booking for the delivery - means that she showed resilience in her approach:

“I went out and sat under that tree for quite some time, listening to the pains. Something came to me that I should go to the clinic, you see. But then I thought that I would go to the clinic but when I get there, they were going to shout at me and accuse me of not booking in time and maybe not even attend me. So I told myself that I will see what I do. If it takes for me to give birth by myself then so be it, because it doesn’t seem like I have a choice.”

When Sthembiso described how she coped on her own with labour pains, she showed resilience based on her beliefs, the strength gained from overcoming previous challenges, and the idea that this was only a temporary issue:

“You know I’m a kind of person that doesn’t really care about stuff. I just told myself that I have been through a lot more than this, big things, which I have been through in my life, and this is just a temporary pain and it will pass.”

Despite having a young infant, Sthembiso hopes to continue going to school. She aims to maintain a positive outlook, opting to consider the addition of her own child to an existing set of difficult life circumstances as not particularly burdensome:

“Well, so far I am coping because having a child of my own is not something that I would say has brought a difference in my life because I have been looking after my sisters for ever and I don’t think I will have difficulty in looking after a child of my own. I don’t know what will happen as time goes on but for now I’m still fine.”

She has thus coped with her circumstances with little help from the health care system. She has resisted becoming a cog in what she perceived for a time to be a very confined system. She did not feel victimized and navigated the arrival of her baby through her resilience aided by her beliefs and supportive neighbours. Her asset base is cultural and strength of character. Yet endurance seems central, although it might lead to unhealthy behaviours or practices.

### Thulani’s story: resistance may be deadly but not futile

Thulani Zondo is a single, unemployed man in his mid-thirties sharing a small house with an older brother. He receives treatment for both HIV and TB. His father died when he was very young, his mother when he was a teenager. He has nine siblings but has little contact with some of them. As a twelve year old and again as an older teenager, he was arrested for petty theft and later for stealing car radios, and his teenage years were marked by long periods in juvenile detention and prison. However, at the time of the interview, Thulani appeared to have distanced himself from his previous criminal life, having first worked in a fast-food outlet and now trying to earn some money by washing cars and selling ice cream. Nonetheless, Thulani described some conflicts with his brother who was using their shared house as a base for some criminal activities.

Thulani has a poor health history with a diagnosis of pneumonia, which continued for three months with noticeable weight loss. His sputum and blood were then tested, revealing TB and HIV infections, and he was put on medications for both. When learning about his diagnosis and early on during his treatment, he felt depressed but later developed a variety of ways to increase his resilience to the challenges of illness. He recognized that he must resist this idea of victimhood and stigmatisation. He remembered:

“I felt hurt but I told myself that I have to be positive. I am still a human being like everybody. Then I decided that I have to tell my neighbor about this. (…) Yes, I was just telling him. Then, the next day, when I was here to collect my treatment and the nurse told me to go to the other room to fetch a certain letter, then I found him sitting down in that room. Then I was very relieved to find that we are on the same boat.”

There were other sources of emotional and spiritual support to enhance his resilience:

“At church I go there because there is a lady that is in the same situation that I am in. She usually comes here and we sit together and advise each other and we also read the bible together…”

Social interactions are however complicated and may diminish resilience unless some of their negative effects are resisted:

“…even though they are not telling me but their actions tell me that they are treating me well. Even though sometimes they do gossip about me. Sometimes you find that when I pass by, people would start asking each other about the symptoms that I have and maybe one of them would say that I suffer from HIV. And some of them when they tell me that they were gossiping about me, I just tell them that that doesn’t bother me because we are all sick.”

There is a sense of enduring difficult interactions through his own agency but that resistance can sometimes compromise health status. Thulani defaulted as he was undertaking work from his home and unable to reach the clinic during opening times. When he returned to treatment, the nurse shouted at him, humiliating him publicly for defaulting.

“One day I came here and I once defaulted [not collecting his treatment for a month]. Then the nurse was standing in front of many people talking to me loudly saying that my CD4 count now is very low and I have to start the treatment afresh. And that hurt me a lot because she should have been polite. Maybe call me to her office because she’s got one, and talk to me nicely. Explain that because I have defaulted and my CD4 count is low, that I have to start the treatment afresh. Unlike what she did, shouting at me in front of many people. Then I went home but I was not ok with that. Then, after two or three days, I came back here and told her how I feel about what she did. And she only shouted back asking me why am I there at that time to collect the pills and that’s when I decided to give up the pills.”

However, after some time without treatment, his health deteriorated. He developed visible sores. After eight months without medication, he decided to forgive the nurse and went back for treatment. Thus resistance in the form of defaulting from treatment was indeed futile and he recognized that to get better and become resilient again requires – in the face of an unyielding system/unrepentant provider - compromise:

“The fact that I got into an argument with the nurse and not counting the fact that I was working, I think that’s what made me default. And the fact that I was going to (have to) start afresh the whole procedure. Then I stopped coming here. But after a while thinking clearly, sitting at home, I realized that I was making myself sick; because I started having some sores on my face and during the night, I could not sleep because I was busy scratching myself. Then that’s when I decided to come back here because I did not change the clinic. I started here and I will continue here. Then I came back here and the nurse welcomed me back even without my apology.”

For Thulani, his refusal to be a victim of inappropriate provider behaviour showed that he was able to cope with the challenges of the health care system as he navigated his somewhat chaotic life and social relationships. But he further realized that not committing to treatment would result in compromised health and possible death. The to and fro of resilience and resistance is shown in his compromise in returning to the clinic and further in his re-acceptance by nursing staff. Their compromise is in viewing his earlier challenge to their expertise as one interaction with a patient who requires care. Furthermore, Thulani is willing now to endure possibly difficult relationships with staff to ensure treatment. Thulani has reshaped his resource base in returning to the clinic and as his story reveals he has few assets in the community in which he lives.

### Nomathemba’s story: resilience for caring

Nomathemba Mxeke is a social worker who assists HIV patients with various issues including counselling, disclosure of HIV status, accessing the disability grant and obtaining food parcels. Her story emphasizes provider interaction with patients to enhance their resilience. Furthermore, her resistance to the controls within the system cost her money as she pays for patients transport costs, when she senses that their treatment adherence might be affected because they are unable to pay for transport to the clinic. In some ways, context is matched by her endurance by acting against the odds she perceives and she develops resilience through several strategies:

“I’m not supposed to do it, you know but … I will issue out my money, you know: ‘go buy, go buy…whatever’ or sometimes I would issue taxi fare, especially to our cases [from a nearby impoverished informal settlement]. A person, a patient will tell you that: ‘I’ve walked to here and I don’t have money.’ And you’ve seen how full our clinic sometimes is, they will sit here all day. Even if I have an apple in my bag, I will, you know, take out my apple and give or I will give five rand taxi fare. Sometimes I even use my car [sighs] to transport them, that’s how far I can go. But I am just helping. Maybe God will reward me one day… That’s how I work sometimes.”

She attempted to resist how the health care system (often in relation to social and welfare development) works by helping patients with appeals for disability grants:

“You find that they don’t know how to write or they don’t want to do it on their own, you know. Then I’ll say, ‘go home, try it. Jot down something. Come back to me, let’s discuss it.’ … But even if you write a motivation, it really doesn’t help because at the end of the day, the doctor decides. It’s their job. You can’t query how they do their job. So normally, I will only assist in terms of writing appeals and then refer to other NGOs that maybe issue out food parcels. And we refer them to join support groups because in other support groups, they offer food parcels. And that’s how I would handle a case. But it’s not easy, hey?”

While she clearly understands the poverty, social conditions and adversities which challenge patient access and at the same time challenge her ability to keep and maintain a professional distance, there is a strong sense of optimism, humour, personal confidence and professionalism which underlies her discourse.

“It becomes very difficult because we do sometimes have patients who will say: ‘I’m on my own, there’s no one, there’s no friends, there’s no relatives. It’s just me and you and the clinic, you know.’…. But we just work with them. They come in here, sometimes even if it’s not their dates to come to the clinic. They will just come and say: ‘we want to see you.’ And then we’ll sit and talk. And talk. And talk. And talk. And talk. Then they go. ‘Okay, now are you fine?’ ‘Yes I’m fine.’, then they go. Sometimes they call me. So I try to be available as much as I can. Though it’s not easy.”

Her resilience is assisted by professional networks of social workers which provide a space for sharing and unburdening the challenges arising from her work environment.

“One way of me getting support is I talk to other social workers and then we meet once in a month whereby we debrief, we discuss our cases. We once tried to form a forum of social workers but because of other issues, it just faded away. But we are trying again to revive it. I don’t know if it will work. But that was another way of helping us to cope and deal with whatever challenges we are facing in this profession and the sites we are working in.”

Her resilience is strengthened by patient acknowledgement which contributes to building her self-esteem and work motivation. She also notes that while being empathetic, she maintains a professional distance from her clients and strives to separate her professional and personal life, although that remains a challenge. These are clearly assets which allow her to cope and build resilience.

But provider resilience often means resisting becoming too attached:

“Not to become attached because they want to talk, they want friends. I think others, they believe that maybe we can be their friends. … We can be their friends, but we don’t have to create that dependency. And when you tell them that: ‘Things are supposed to be like this, not like that’, they become very disappointed. But, anyway, it’s like that.”

And her resilience is compromised by the slow rate of learning about and acceptance of HIV/AIDS:

“I think challenges they present to me or for me would be the ignorance, I think, around HIV/AIDS. And having to know that we have had this thing for so many years and it’s not going to go away any time soon. But you still find employers or families or couples … relationships where people are still discriminating other people. Discriminating other people, calling them names, judging them, treating them differently. Some times when a patient comes here and says: ‘At work they are doing one, two, three’, I feel like picking up the phone and screaming at the employer. But I try to calm down myself first so that I can think reasonably and then I will advise myself, sit down, calm, sometimes.”

But enduring these challenges is necessary and helps her bounce back:

“We’ll get patients who’ll say to you, ‘I don’t know why I was sent here’. And others will be: ‘No, they made a mistake. I’m not HIV positive, you know.’ And also having to deal and accept their situation the way that it is. So I think maybe as professionals also having to explain, why we are taking these tests, what is it now that you have to do? And then others, they will just say: ‘they didn’t explain that I have to start ARVs. Yes, I know my status but I was just sent here. Maybe to start your prophylaxis but they didn’t tell me anything about starting ARV classes or something like that’. So maybe also, explaining to our patients on our side. ‘Now that you have tested, these are the steps you have to do. Are you okay with it? Or maybe go home and think about it? … Then, when the person is ready, they will come here. Maybe that will also decrease our defaulter rate as well.”

Nomatheba’s story points to provider resistance to how the system operates, trying at the margins to make it more personable and responsive. She subverts some elements of control, especially the often dysfunctional allocation of assistance, by using her own money and engaging NGOs. Her resource base is financial and professional. She can help her patients endure the conditions in which they live and maintain adherence to treatment. She also challenges her patients’ and society’s ‘ignorance’ of the disease, gaining satisfaction and the ability to continue from her own motivations and professional support.

### Grace’s story: resistance for caring

Grace Themba is a 26 year old pharmacy assistant who has been employed at the clinic for 2 years. She speaks of the challenges of a heavy patient load and working in a resource-constrained environment with insufficient doctors and pharmacists.

She, too, emphasizes trying to enhance the resilience of patients in downplaying their victimhood in a situation of resource scarcity and trying to provide clear instructions, often leading to repetitive work:

“Some of them - we tell them how to take [the medication], but when they go home they come back, they didn't do it correctly – so we have to sit down again, which is done by the counsellors who… do pill counting, the process is done by doctors, and also I have to make sure that I do it again.”

Respect and patience for the patients – to enhance their ability to express challenges and to cope with painful illnesses - is also important to maintain treatment adherence:

“They come in, we close the door. We only see one patient at a time, so they are able to express how they feel. So what I do, I can always take the file, go back to the doctor with the patient and explain that this is the problem that's happening to one of my clients. She was afraid to tell [the doctor, but] then they sit down and discuss it.”

Her own resilience is ensured by teamwork and meetings to discuss issues:

“And then if it's a Monday … we have a meeting … we discuss our problems that we experienced around the clinic the previous week and how to deal with the problems in the future, we give reports. If I'm short of treatments I tell them that “I don't have this, I don't have that, but I have this you can use it” and then blah-blah-blah, what's happening in the pharmacy, they also keep their reports if there's any problems with us, the pharmacy, they also tell us that we like you to do things this way.”

Her attitude to her patients has changed:

“Before I started working here, I thought HIV was this dreadful disease and here at the clinic people are always sick, they are always on the floor, everything. I thought it was chaos. But then I started working here and I saw that people are living positively. You won't even recognize that they're HIV positive. So that's our problem we have… we have little knowledge when we are outside. So I'll just bring them here to see the environment and see how people are living. They don't have any problems, especially our patients they… they've already accepted their status so … everything is fine. You can see it in their faces.”

Thus far, Grace’s story is similar to Nomathemba’s, emphasizing the enduring and resilient qualities of patients. But part of her story about herself more strongly emphasizes resistance to system operation. She comments on the paucity of service delivery, which emphasizes the importance of respect for patients and resisting the notion that they are just clinic card numbers:

Patients “have to wait outside. Yes, they get seen late. They don't have a dietitian there. There's no doctor, only nurses. They don't have a social worker so… and their space is… just one room. I can come and dispense my medication there. I'm sitting with you, you're my patient, and a nurse is sitting over there with another patient. So they can't explain their problems, what they are experiencing either at home or visiting – they are afraid to do that because another patient is sitting over there and eavesdropping.”

She continues:

“There are no doctors, there are no pharmacists. In fact… it's a chaos especially here in our hospitals, we don't have staff. People, they queue until late in the afternoons. When you come at night, there's no doctor on call. People [providers] are leaving this place, people are leaving and it's a serious issue. Nobody's taking it for serious but it's really serious here.”

Off tape, she commented, as a form of resistance, that there are too many managers and not close scrutiny of how resources are spent. These are themes common in recent reviews of the system. Yet Grace enjoys and is committed to her work, relationship with patients and is confident that patients value the care that they receive at her clinic. This motivates her. She also draws strength from the positive and supportive relations with colleagues, which she likens to “feel[ing] like home”. These assets build her resilience and override feelings of “helplessness” arising from patients who do not adhere to treatment and ignore her advice and recommendations. And they also in some way compensate for her perceptions of management. It should, however, be noted that on returning to the clinic, we were told that Grace had left to work in an urban area and also further her education. It is perhaps likely that as well as this being a decision to improve her own circumstances, it was made easier by the difficulty of resisting poor resource allocation and conditions in which patients are provided with few amenities and little respect.

## Discussion

### Contextualizing the stories

These four cases illustrate the complex interactions in the South African health care system which itself is constrained in its operation by the economic and political context of the country (see [8, 10, 12]). It is perhaps these contexts which lead to feelings of forbearance and endurance among many patients and providers. We note in these stories many of the now commonplace ideas about the health care system - issues of disclosure, problems of compliance and adherence to treatment, discourses of domination and patient passivity, victimhood and vulnerability. But individuals must cope with and adapt to circumstances and the systemic barriers to accessible care. They use their variable resources not just to endure but to resist and become resilient given their life conditions. All four cases reflect endurance. But they also reflect resistance or resilience, depending on circumstances shaped by their individual and social context and the role of agency. In discussion, these relationships are underscored with reference to other respondents and in the light of the theoretical lenses of coping and agency.

Resistance entails a challenge to the status quo in attempts to protect one’s self and values [29]. The stories presented note these attempts but they are not alone. Other patient respondents emphasize these ‘weapons of the weak’ [51], including withdrawing or saying no to provider demands. So Naledi, an HIV-positive patient, whose resistance may place her own health at a disadvantage, says:

“Although I arrived at 11h45am the nurse told me, in front of everyone, where to get off because I was late on that day and she complained that this affected how they assemble their card system. So because I am ‘late’ she said I should come back on Wednesday. But I thought clinics operate until 4 pm. I also told them that I am not going to come back there and if anything happens to me, she is going to be responsible for that.…I also concluded that she is going to be responsible for anything that can happen in my life and now I have stopped taking pills completely.”

For patients with few assets, it becomes particularly difficult to resist a system which seems to be organized to suit staff needs rather than those of patients. They must endure dead time while waiting for treatment, arrogant comments, and perceptions/expectations of the health system and health care providers, adding stress to their challenging life circumstances. This is not uncommon and patients may pick up on media portrayals of poor health care practice to demonstrate perhaps a futility to resistance and the need for stoicism, perhaps itself a form of resistance. “The staff asked if they could do a post mortem but she said (bristling), “no way, you already told us why the baby died – now you just want to steal the heart and kidneys for someone else”“(observation notes, Johannesburg)

For providers, resistance was in part concerned with how the health care system operated as well as attending to the demands of ignorant patients. Other providers blamed patients, feeling that they (the patients) have too many rights, perhaps an implicit view of how post-apartheid changes, including the formalisation of patients’ rights in law and policy, have affected the provider-patient relationship. Some tell patients that defaulting will result in death with the patient to blame.

“I hate the patients’ rights with all my heart.... Before, patients were taught, patients were nursed, patients were given treatment. Patients were patients. Today, they want to tell us what to do… And you can’t do a thing, your hands are tied. It’s his constitutional right. I think they should be removed or monitored at least.” (TB nurse, Johannesburg)

System operations and the rights agenda of the democratic era lead some providers to ‘punish’ patients even more. Patients’ rights – now encoded and formalized - continue to be challenged and resisted by providers, adding to the burden placed on patients as well as to the providers themselves.

Resilience is a process in which various resources are used to respond to the adversities of illness or ineffective health care organization* [30]. For patients, those resources can be their belief system, family or community support, or new knowledge gained:

“[My mother] is taking care of me… I am here today because of her. If I need something, I just tell her. Like yesterday, she went to receive her grant. She bought me a small bag of mealie meal and meat.” (Mvelo, patient, Bushbuckridge.)

“I read a lot of pregnancy books just to know more and also because of my HIV status as a pregnant woman and the fact that I must put extra efforts. …And with my boyfriend’s support, I started getting over that anxiety and worry about the implications of being HIV positive and pregnant. So when I went to the clinic, they would explain these things like taking blood tests and booking for delivery in time and I already knew these things” (Gugulethu, stillbirth, Johannesburg).

Provider resilience is enhanced through helping patients and working with committed teammates. Plans are developed to ease patient burden and treat them with respect:

“We actually came up with a strategy to say if the person has come for the first time, we have seen the condition of the patient and they are still ok. And this person is working, we are saying ‘can you send someone else to get your TB treatment every two weeks’?” (TB-nurse, Johannesburg)

The provider narratives also usually recognized the importance of keeping going, of bouncing back and of enduring any dysfunctional elements in the system for the sake of the dignity of patients:

“And then you are losing a patient …And there is nothing you can do. You just sit down, and look at somebody dying. Nothing you've been able to do. But it's sad. It's sad, but what do you do? You have to continue helping others. And just allow that to go by. It's difficult, but you just have to carry on.” (Jamar, Registrar, Johannesburg)

From our four narratives and these supporting cases, resistance, resilience and endurance describe not only the actions and interrelationships of patients and providers in a complex, health system but also provide a sharper lens on coping with or without a strong resource base. In many ways, we assert, endurance is the bedrock of coping, requiring making do and performing the roles required. Endurance understood in this way is not merely passivity but rather permits emotion and problem-based coping. Like resistance and resilience, it is an expression of agency, negotiated and mediated through individual expectations, interpersonal engagements and the system itself. But individuals – and this appears in their stories – alter their views and practices. For themselves as patients or providers, the monolithic, difficult-to-change health care system means a struggle to continue. To navigate within this system, they may engage in individualised, micro-practices of resistance or resilience. A nuanced set of relationships exists between these practices – they shift and flow, dependent upon specific interactions and locales, ways of life and systemic operational constraints. Indeed although there are limitations to this study in terms of generalizability, bound as it is to individual stories told at one point of time and within a particular resource-poor setting, there is a contribution to how individuals use their capabilities [27, 28] and assets [31] through shaping and reshaping their life conduct (i.e. agency) [24] – within *this* very place and time - to cope and even more, to deal with demands and opportunities of everyday life.

## Conclusion

In this paper, we have taken a specific view on how patients and providers cope with the practices inherent in the health care system and with each other’s behaviours. We have used ‘endurance” (almost the steady state of coping), ‘resistance’ and ’resilience’ to demonstrate the adaptive power of individuals in trying circumstances to deal with difficult illness or treatment settings. Although these are individualized responses, they do not diminish the importance of systemic forces, deriving from values and historic ways of acting in the public health system. Furthermore, as told in the four cases, endurance, resistance and resilience, victimhood and adaptation, context and agency are all intertwined. As coping mechanisms, endurance, resistance and resilience are responses to context. They are ‘weapons of the weak’ (see [53]) or ‘tactics’ (see [52]) for manoeuvring, managing and contesting power in systems. They represent ways through which illness and occupation can be made personally meaningful as they make personal identities vivid. Resistance may be in the back spaces of behaviour (see [52]) but it does have the potential to alter the ways in which the health system works, especially if citizen engagement is taken forward as laid out in the National Development Plan (see [7]). These weapons – denial and refusal to behave as expected, recognizing human dignity in the most trying of circumstances – further resilience. Resilience (enduring life conditions, bouncing back and building on meaningful, supporting partnerships) can in itself enable individuals to live with inequities, to endure and survive, and to maybe fight for positive change. Such agency might lead to system improvement as a partial journey to system ‘transformation’. Learning from the problems of endurance (which reveal system-level constraints and impositions), resistance (which can be self-harming) and resilience (individual adaptations that may have system relevance), there are ways of improving the practice and receipt of care. Yet with complex, often chaotic lives of patients and providers, this transformation and these improvements require provincial and national leadership and the will to instigate the admirable reforms set out in policies for the health care system.

## References

[1] Mayosi BM, Lawn JE, van Niekerk A, Bradshaw D, Abdool Karim SS, Coovadia HM (2012). Health in South Africa: changes and challenges since 2009. Lancet.

[2] Cooper D, Morroni C, Orner D, Moodley J, Harries J (2004). Ten years of democracy in South Africa: documenting transformation in reproductive health policy and status. Reprod Health Matters.

[3] Penn-Kekana L, Blaauw D, Schneider H (2004). ‘It makes me want to run away to Saudi Arabia’: Management and implementation challenges for public financing reforms from a maternity ward perspective. Health Policy Plan.

[4] London L, Holtman Z, Gilson L, Erasmus E, Khumalo G, Oyedele S (2006). Operationalising health as a human right: monitoring tools to support implementation of the patients’ rights charter in the health sector.

[5] Gilson L, Daire J: Leadership and Governance within the South African Health System. SAHR 2011, 70–80

[6] South African Human Rights Commission (2010). Public Inquiry: Access to Health Care Services.

[7] Republic of South Africa (2012). National Development Plan: Our Future: Make it Work.

[8] Mangcu X (2012). Rethinking Africa's political economy: an institutionalist perspective on South Africa. Dev.

[9] Alexander P (2010). Rebellion of the poor: South Africa’s service delivery protests – A preliminary analysis. Rev Afr Polit Econ.

[10] Matsosoi MP, Strachani B: Human resources for health for South Africa: HRH Strategy for the health sector 2012/13 – 2016/17. In South African Health Review 2011. Edited by Padarath A, English R. Durban: The Health Systems Trust; 2011: 48–58. http://www.hst.org.za/sites/default/files/Chap%204%20HRH%20Strategy%20pgs%2049-58.pdf

[11] Fried J (2008). The community health worker programme as a response to HIV/AIDS in South Africa: a case study on oNompilo in KwaZulu-Natal.

[12] Ataguba JE, Alaba O (2012). Explaining health inequalities in South Africa: A political economy perspective. Dev Southern Africa.

[13] Barchiesi F (2011). Precarious liberation: workers, the state, and contested Social Citizenship in postapartheid South Africa.

[14] Lazarus RS, Folkman S (1984). Stress, Appraisal and Coping.

[15] Skinner E, Edge K, Altman J, Sherwood H (2003). Searching for the structure of coping: A review and critique of category systems for classifying ways of coping. Psychological Bulletin.

[16] Lee JKL (2003). Job stress, coping and health perceptions of Hong Kong primary care nurses. Int J Nurs Pract.

[17] Dennis C-L (2003). Peer support within a health care context: a concept analysis. Int J Nurs Stud.

[18] Johnson MO (2011). The shifting landscape of health care: toward a model of health care empowerment. Am J Public Health.

[19] Yeji F, Klipstein-Grobusch K, Newell M-L, Hirschhorn LR, Hosegood V, Bärnighausen T (2014). Are social support and HIV coping strategies associated with lower depression in adults on antiretroviral treatment? Evidence from rural KwaZulu-Natal, South Africa. AIDS Care.

[20] Pappin M, Wouters E, Booysen FLR (2012). Anxiety and depression amongst patients enrolled in a public sector antiretroviral treatment programme in South Africa: a cross-sectional study. BMC Public Health.

[21] Rosenberg C (2009). Managed fear. Lancet.

[22] Giddens A (1982). Profiles and Critiques in Social Theory.

[23] Folkman S, Lazarus RS (1988). Coping as a mediator of emotion. J Pers Soc Psychol.

[24] Jungar K, Oinas E (2011). Beyond agency and victimisation: re-reading HIV and AIDS in African Contexts. Soc Dynam.

[25] Cockerham WC, Abel T, Lüschen G (1993). Max Weber, formal rationality and health lifestyles. Sociol Q.

[26] Hays S (1994). Structure and agency and the sticky problem of culture. Sociol Theor.

[27] Sen A, Nussbaum M, Sen A (1993). Capability and well-being. The Quality of Life.

[28] Abel T, Frohlich KL (2012). Capitals and capabilities: Linking structure and agency to reduce health inequalities. Soc Sci Med.

[29] Pardo del Val M, Martinez Fuentes C (2003). Resistance to change: a literature review and empirical study. Manage Decis.

[30] Fraser M, Richman J, Galinsky M (1999). Risk, protection, and resilience: toward a conceptual framework for social work practice. Soc Work Res.

[31] Obrist B, Pfeiffer C, Henley R (2010). Multi-layered social resilience: a new approach in mitigation research. Prog Dev Stud.

[32] Afana A-H, Pedersen D, Rønsbo H, Kirmayer LJ (2010). Endurance is to be shown at the first blow: Social representations and reactions to traumatic experiences in the Gaza Strip. Trauma: Int J.

[33] Fried J, Harris B, Eyles J (2012). Hopes interrupted: accessing and experiences of antiretroviral therapy in South Africa. Sex Transm Infect.

[34] Thomas G (2011). A typology for the case study in social science following a review of definition, discourse and structure. Quali Inq.

[35] Mills CW (1959). The Sociological Imagination.

[36] Bamberg MGW, McCabe A (1998). Editorial. Narrat Inq.

[37] Skultans VL (1999). Narratives of the body and history: illness in judgement on the Soviet Past. Sociol Health Illn.

[38] Waring J, Currie G (2009). Managing expert knowledge: organizational challenges and managerial futures for the UK medical profession. Organ. Stud.

[39] Waring J, Bishop S (2010). Lean healthcare: Rhetoric, ritual and resistance. Soc Sci Med.

[40] Eyles J, Donovan J (1990). The Social Policy of Health.

[41] Mishler E (1999). Storylines: Craft artists' Narratives of Identity.

[42] Riessman CK (1990). Strategic uses of narrative in the presentation of self and illness. Soc Sci Med.

[43] Morse JM, Field PA (1995). Qualitative Research Methods for Health Professionals.

[44] Crowe S, Cresswell K, Roberston A, Huby G, Avery A, Sheikh A (2011). The case study approach. BMC Med Res Methodol.

[45] Mattingly C, Garro LC (2000). Narrative and the Cultural Construction of Illness and Healing.

[46] Goffman E (1969). Strategic Interaction.

[47] Creswell J, Hanson WE, Clark Plano VL, Morales A (2007). Qualitative research design: selection and implementation. Couns. Psychol.

[48] Yin R (2011). Qualitative Research from Start to Finish.

[49] Twigg J, Wolkowitz C, Cohen RL, Nettleton S (2011). Conceptualising body work in health and social care. Sociol Health Illn.

[50] Geertz C: After the Fact: Two Countries, Four Decades, One Anthropologist. Cambridge: Harvard University Press; 1995

[51] Scott J (1985). Weapons of the Weak: Everyday Forms of Peasant Resistance.

[52] Scott J (1990). Domination and the Arts of Resistance.

[53] Schneider H, le Marcis F, Grard J, Penn-Kekana L, Blaauw D, Fassin D (2010). Negotiating care: patient tactics at an urban South African hospital. J Health Serv Res Policy.

